# Plasma and Urine Metabolites Associated With Nondiabetic Chronic Kidney Disease: The HELIUS Study

**DOI:** 10.1016/j.xkme.2025.101009

**Published:** 2025-04-17

**Authors:** Charlotte M. Mosterd, Barbara J.H. Verhaar, Bert-Jan H. van den Born, Max Nieuwdorp, Daniel H. van Raalte

**Affiliations:** 1 Department of Internal and Vascular medicine, Amsterdam University Medical Centers, AMC, Amsterdam, The Netherlands; 2 Diabetes Center, Department of Internal Medicine, Amsterdam University Medical Centers, VUmc, Amsterdam, The Netherlands; 3 Department of Public and Occupational Health, Amsterdam University Medical Centers, Amsterdam, The Netherlands

**Keywords:** Chronic kidney disease, metabolomics, machine learning, ethnicity

## Abstract

**Rationale & Objective:**

We aimed to find predictive plasma and urine metabolites for nondiabetic chronic kidney disease (CKD), and to validate these biomarkers in a diabetic kidney disease (DKD) population, using data of the population-based multiethnic Healthy Life in an Urban Setting study.

**Study Design:**

Cross-sectional metabolome study.

**Setting & Participants:**

From the Healthy Life in an Urban Setting population-based cohort, we included 124 participants with nondiabetic CKD, 45 with DKD and 200 healthy controls.

**Predictors:**

Plasma and urine metabolites were measured using ultra-high-performance liquid chromatography coupled to tandem mass spectrometry (LC-MS/MS) with an untargeted approach.

**Outcomes:**

(Nondiabetic) CKD.

**Analytical Approach:**

We used machine learning models to predict nondiabetic CKD from metabolite profiles and used logistic regression models with adjustment for potential confounders to verify our results in the best predicting metabolites. In addition, we assessed the associations between the best predicting metabolites and DKD.

**Results:**

Urine metabolites were more predictive of nondiabetic kidney disease than plasma metabolites. In plasma, the best predicting metabolites for nondiabetic CKD included many amino acids, including N-acetylated amino acids, histidine, and indolepropionate. In urine, the highest-ranked metabolites were predominantly lipids, including sphingomyelins and phosphatidylcholines. There was limited overlap among the top-ranked metabolites in predicting nondiabetic CKD between plasma and urine. Almost all associations with nondiabetic CKD could be translated to DKD. No interactions were observed with ethnicity.

**Limitations:**

The cross-sectional design limits causal inference.

**Conclusions:**

Our analyses revealed that urine metabolites were strongly associated with CKD than plasma metabolites in this multiethnic population. The finding that specific associations of plasma and urine metabolites could be translated to subjects with DKD suggests a shared pathophysiological background.

## Plain Language Summary

Chronic kidney disease (CKD) has a rising incidence, yet its underlying causes are not fully understood. Using the multiethnic Healthy Life in an Urban Setting study, we explored which molecules in blood and urine (metabolites) are different in patients with CKD with albuminuria and preserved estimated glomerular filtration rate. Urine metabolites, particularly lipids like sphingomyelins, were more strongly associated with CKD than plasma metabolites, which included amino acids such as histidine and indolepropionate. These findings were also applicable to patients with diabetic kidney disease, suggesting shared disease mechanisms. Our study suggests that metabolomics may help identify metabolic changes linked to CKD and DKD and sheds new light on potential pathogenic pathways.

The worldwide incidence of chronic kidney disease (CKD) has increased sharply over the past few decades and is foremost driven by the rising prevalence of conditions, such as obesity, and hypertension, type 2 diabetes (T2D).^1^^,^^2^ When CKD is present in people living with diabetes, it is referred to as diabetic kidney disease (DKD). Although hyperglycemia may induce a pattern of well-described glomerular lesions, the pathological lesion observed in people with T2D, who often suffer from comorbid conditions that put the kidneys at risk, are much more heterogenous and display strong overlap with nondiabetic CKD.^3^^,^^4^

Both CKD and DKD are not evenly distributed among the population, as prevalence significantly varies among different ethnic groups.^5^ In the Amsterdam-based Healthy Life in an Urban Setting (HELIUS) study, for example, prevalence of CKD was higher in South Asians and individuals of sub-Saharan African descent and consistent with the higher prevalence of kidney risk factors.^6^^,^^7^ This underlines the importance of a diverse study population when investigating CKD.

In recent years, novel treatment options have emerged for the treatment of CKD and DKD that have been shown to improve kidney outcomes.^8^ However, what is also clear from the large outcome trials that were conducted is that residual risk for CKD and DKD progression to kidney failure remains high, likely due to the fact that pathophysiological pathways remain incompletely understood. Thus, detailed understanding of disease mechanisms is pivotal to prevent or to reduce progression toward kidney failure in these individuals.^9^

Biopsy studies have generated a wealth of data allowing to dissect disease mechanisms, but it’s use remains limited due to its invasive nature. Untargeted blood or urine metabolomics is a powerful technique to identify novel metabolic pathways contributing to disease etiology. In this study, we examined which plasma and urine metabolites are associated with nondiabetic CKD in the multiethnic Healthy Life in an Urban Setting (HELIUS) Study, using machine learning prediction models. In addition, we determined whether these metabolites associated with nondiabetic CKD are specific to this condition or also translate to individuals with DKD.

## Methods

### Study Population

We used data from the HELIUS study, a large, multiethnic cohort in Amsterdam, The Netherlands, with data collected at baseline visits (2011-2015). Detailed study design and methodology have been published previously.^10^ Participants aged 18 to 70 years old were randomly sampled, stratified by ethnicity, based on the Amsterdam municipality registry. The study received approval from the medical ethical review board of Amsterdam UMC, and all participants provided written informed consent.

Before the study visits, participants were asked to refrain from smoking. Body mass index (BMI) was calculated from height and weight, and blood pressure was measured twice in a supine position using a validated device, with hypertension defined as previous diagnosis, blood pressure-lowering medication, or blood pressure > 140/90 mm Hg.^11^ Fasting blood samples were collected to measure glucose, creatinine, and eGFR (CKD-EPI formula). Urinary albumin-to-creatinine ratio (UACR) was measured from early morning urine samples; albuminuria was defined as UACR ≥3 mg/mmol following KDIGO guidelines.^12^ Diabetes was defined by a history of diabetes, fasting glucose > 7 mmol/L, or glucose-lowering medication. The HbA1c levels were measured via high-performance liquid chromatography. Ethnicity was determined based on self-report.

For this analysis, we selected 124 participants from the HELIUS cohort (N = 22,165) with nondiabetic CKD (defined as albuminuria KDIGO stage A2, UACR 3-30 mg/mmol), preserved eGFR (> 60 mL/min/1.73m^2^) and available plasma and urine samples, while excluding those with diabetes. The flowchart of the selection process is shown in Fig S1. This group included South Asian Surinamese, Ghanaian, African Surinamese, and European Dutch participants. Controls were 200 randomly selected participants (50 from each ethnicity) without diabetes or CKD, and with normal kidney function (UACR < 3 mg/mmol, eGFR > 60 mL/min/1,73m^2^).^12^ There was no data available on underlying etiologies. From an epidemiological point of view, glomerulosclerosis (hypertension-related), obesity-associated CKD, and IgA nephropathy (relatively common in The Netherlands) are the most likely causes of CKD in this study population. For secondary analyses, we assessed associations in 45 participants with DKD (with the same reference controls), defined as the combination of albuminuria and diabetes.

### Metabolomics

Fasting plasma (n = 369) and urine (n = 368) samples were frozen at −80 °C and analyzed by Metabolon using untargeted metabolomics. Recovery standards were added pre-extraction for quality control, with protein precipitation achieved in methanol, centrifuged, and prepared for various ultra-performance liquid chromatography (UPLC) methods. Each batch included quality control controls, pooled plasma, and spiked standards to monitor instrument performance and alignment. Samples were analyzed on a Waters ACQUITY UPLC paired with a Thermo Scientific Q-Exactive mass spectrometer (35,000 mass resolution; 70-1000 m/z range). Reverse-phase and HILIC columns (C18 and BEH Amide, respectively) were used. Compounds were identified by comparison to a library of purified standards based on retention index, mass match (±10 ppm), and MS/MS scores. Missing values were imputed as the lowest observed values. After excluding xenobiotics, datasets included 722 plasma and 759 urine metabolites, with urine normalized to creatinine. Full UPLC-MS/MS details are in the Item S1.

### Machine Learning Models

We used machine learning classification models to predict nondiabetic CKD from plasma and urine metabolite profiles. These XGBoost machine learning models had a nested cross-validation design and 200 iterations.^13^ With each iteration, the dataset was randomly divided over a train (80%) and test (20%) set. Within the training set, model hyperparameters were tuned using 5-fold cross-validation. Two random variables were introduced in each iteration to serve as benchmarks. The resulting model was evaluated on the test set, using the area under the receiver-operator curve (AUC) as the primary metric. To evaluate the tendency of the model to overfit, we ran identical models in which all data were permuted before each iteration. Each iteration also produced a ranked list of metabolites based on their relative importance in the prediction. These lists were recorded and averaged across all 200 iterations. The top 20 metabolites with their feature importance (based on their average gain in the model) were plotted, with the highest-ranked metabolite set at 100% and the other metabolites shown relative to this metabolite. The machine learning script was coded in Python (v.3.8.6) using the XGBoost (v.1.2.0), numpy (v.1.19.2), pandas (v.1.1.4), and scikit-learn (v.0.23.2) packages.

### Statistical Analysis

Differences between nondiabetic CKD and healthy controls and between individuals with CKD and DKD were tested with *t* tests for continuous variables with normal distributions, Mann-Whitney U tests for continuous variables with non-normal distributions and χ^2^ tests for categorical variables. We used logistic regression models to determine effect sizes for the associations of the top 20 highest-ranked plasma and urine metabolites resulting from the machine learning models. The metabolites were the determinants in these analyses, and nondiabetic CKD was the binary outcome. In the adjusted models, we included age, sex, BMI, and hypertension status as covariates. Metabolite concentrations were log10-transformed and then standardized to a mean of 0 and standard deviation (SD) of 1. Associations of each metabolite for nondiabetic CKD were plotted in a forest plot showing the odds ratios (OR) per SD increase with the 95% confidence intervals. We tested interactions with ethnicity to assess if these associations were robust across ethnic groups. To assess if the associations were specific for nondiabetic CKD, we used logistic regression models with the same metabolites and DKD as outcome in the study population with control and DKD subjects. All statistical tests in these regression models were corrected for multiple testing (Benjamini-Hochberg). *P*-values considered significant < 0.05. The statistical analyses and data visualizations were conducted in RStudio (v.2022.7.2.576) using R (v.4.2.1).

### Data and Code Availability

The HELIUS clinical and metabolomics data are owned by the Amsterdam UMC, located at AMC in Amsterdam, The Netherlands. Any researcher can request the data by submitting a proposal to the HELIUS Executive Board as outlined at www.heliusstudy.nl/en/researchers/collaboration. The board will check proposals for compatibility with the general objectives, ethical approvals, and informed consent forms of the HELIUS study. Access is granted to all researchers affiliated with an internationally recognized research institution requesting use of HELIUS data after signing the data transfer agreement. All programming code was made publicly available in a GitHub repository (https://github.com/barbarahelena/ckd-metabolomics).

## Results

### Population Characteristics

Characteristics of 324 control and nondiabetic CKD participants from the HELIUS cohort are shown in Table 1. Sex, eGFR, and ethnicity were comparable between individuals with nondiabetic CKD and controls. Individuals with CKD had a higher BMI and systolic and diastolic blood pressure than the control group (*P* < 0.001) and the prevalence of hypertension was in individuals with CKD was higher. Total cholesterol and low-density lipoprotein levels were comparable between the groups, whereas triglycerides were higher in patients with CKD (*P* < 0.001).

**Table 1:** Participant Characteristics

	Controls	Nondiabetic CKD	*P*
N	200	124	
Age (y)	49.6 ± 11.4	52.1 ± 11.0	0.046
Women	109 (54.5)	68 (54.8)	>0.99
Ethnicity			0.575
Dutch	50 (25.0)	34 (27.4)	
South Asian Surinamese	50 (25.0)	24 (19.4)	
African Surinamese	50 (25.0)	37 (29.8)	
Ghanaian	50 (25.0)	29 (23.4)	
BMI in kg/m^2^	26.2 ± 4.5	28.6 ± 5.7	<0.001
Current smoking	33 (16.6)	24 (19.4)	0.627
Hypertension	89 (44.5)	87 (70.2)	<0.001
Systolic BP in mm Hg	130.0 ± 17.7	141.8 ± 22.6	<0.001
Diastolic BP in mm Hg	81.0 ± 10.2	86.5 ± 13.3	<0.001
eGFR in mL/min/1.73m^2^	98.5 *±* 17.1	93.3 *±* 20.0	0.014
UACR in mg/mmol	0.3 (0.2-0.4)	5.4 (93.7-8.4)	<0.001
HbA1c in mmol/mol	37.6 ± 4.6	39.5 ± 4.6	<0.001
Total cholesterol in mmol/L	5.1 ± 0.9	5.1 ± 1.0	0.682
LDL in mmol/L	3.2 ± 0.8	3.2 ± 0.9	0.762
Triglycerides in mmol/L	0.7 (0.6-1.0)	1.0 (0.6-1.5)	<0.001

*Note:* Data are presented as mean ± SD, median [IQR], or n (%). Group differences were tested with *t* test for continuous variables with normal distribution, Mann-Whitney U for continuous variables with non-normal distribution, and χ^2^ tests for categorical variables.

Abbreviations: BMI, body mass index; BP, blood pressure; CKD, chronic kidney disease; eGFR, estimated glomerular filtration rate; LDL, low-density lipoprotein; UACR, urine albumin creatinine ratio.

### Associations Between Plasma Metabolites and Nondiabetic CKD

The classification model to predict nondiabetic CKD from metabolite profiles yielded an AUC of 0.59 (95% CI, 0.58-0.60). The top 20 highest-ranked metabolites in nondiabetic CKD predominantly comprised amino acids and lipids (Fig S2). N−acetylalanine, a derivative of alanine, was the highest-ranked amino acid, followed by formiminoglutamate, an amino acid in the histidine metabolism pathway. Other prominent amino acids included 1-carboxyethylphenylalanine, a derivative of phenylalanine, and histidine. In addition, there were several phosphatidylethanolamines among the top 20 predictors, including 1-palmitoyl-2-oleoyl-GPE (16:0/18:1).

**Figure 1:**
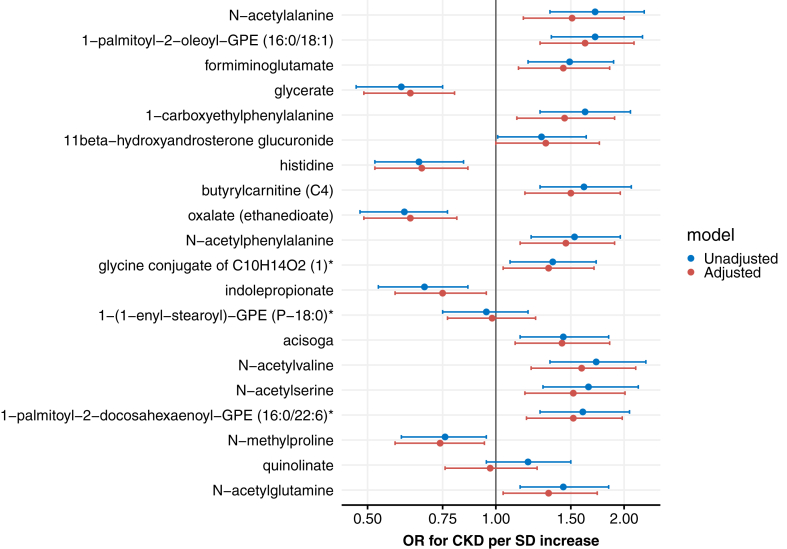
Logistic regression models: odds ratios (OR) per standard deviation (SD) increase of the top 20 plasma metabolites for nondiabetic chronic kidney disease (CKD). In the adjusted model (red), we corrected for age, sex, body mass index, and hypertension.

Next, we used logistic regression models to obtain effect sizes of the associations between the top 20 highest-ranked metabolites and CKD, while adjusting for relevant confounders such as age, sex, BMI, and hypertension (Fig 1; Table S1). Seventeen of the top 20 plasma metabolites resulting from the machine learning model were significantly associated with nondiabetic CKD in the adjusted models after multiple testing correction. Most metabolites were associated with higher odds of CKD. The lipid 1-palmitoyl-2-oleoyl-GPE (16:0/18:1) had the largest effect size with an OR of 1.62 (1.27-2.11, *q* = 0.0026) for CKD with each SD increase after adjustment for confounders. In addition, the amino acids N-acetylvaline (OR 1.59 [1.21-2.13], *q* *=* 0.005), N-acetylserine (OR 1.52 [1.18-1.98], *q* = 0.006), N-acetylalanine (OR 1.51 [1.16-2.00], *q* = 0.006), and N-acetylphenylalanine (OR 1.46 [1.14-1.90], *q* = 0.007) showed strong associations with CKD after adjustment. Four metabolites were significantly associated with lower odds of CKD in the adjusted models, including glycerate (OR 0.63 [0.49-0.80], *q* = 0.003), histidine (OR 0.67 [0.52-0.86], *q* = 0.005), oxalate (OR 0.63 [0.49-0.81], *q* = 0.003), and indolepropionate (OR 0.75 [0.58-0.95], *q* = 0.028). Of note, there were no significant interactions between the top 20 plasma metabolites and ethnicity.

### Association Between Urine Metabolites and Nondiabetic CKD

The classification model to predict nondiabetic CKD from urine metabolite profiles performed better than the plasma metabolite model, with an AUC of 0.65 (95% CI, 0.64-0.66). The 4 highest-ranked metabolites were lipids, such as palmitoyl sphingomyelin (d18:1/16:0), 2 phosphatidylcholines, and the triacylglycerol 1−stearoyl−2−arachidonoyl−GPC (18:0/20:4) (Fig S3). The highest-ranked 20 metabolites also included several amino acids, such as N-acetylglycine, proline, 4-methyl-2-oxopentanoate, and 3,4-dihydroxyphenylacetate. In addition, there were 3 carbohydrates among the best predictors, including 3’-sialyllactose, glucuronate, and ribitol.

**Figure 2:**
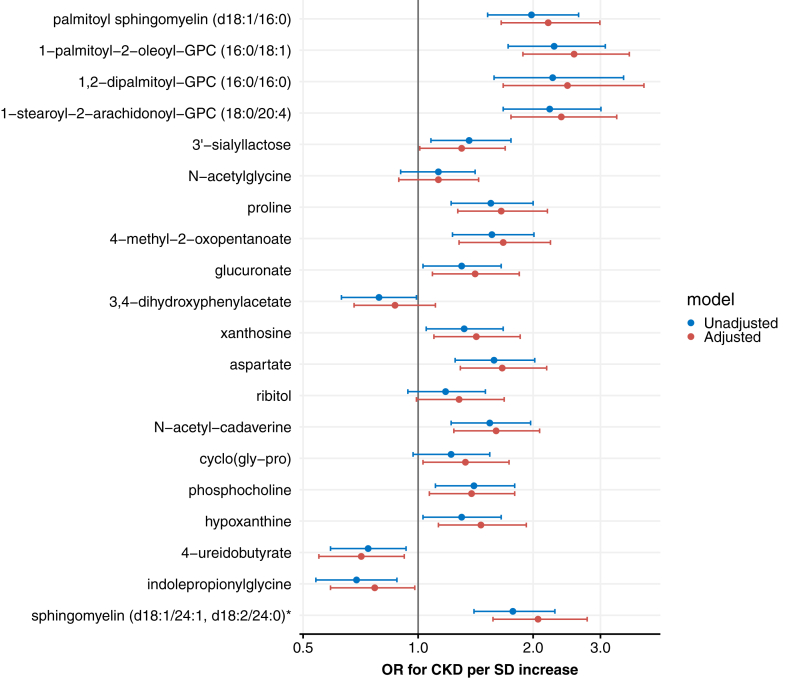
Logistic regression models: odds ratios (OR) per standard deviation (SD) increase of the top 20 urine metabolites for nondiabetic chronic kidney disease (CKD). In the adjusted model (red), we corrected for age, sex, body mass index, and hypertension.

In the fully adjusted logistic regression model, the lipid related metabolites had the largest effect sizes for nondiabetic CKD (Fig 2; Table S2). 1-palmitoyl-2-oleoyl-GPC (16:0/18:1) showed the strongest association (OR 2.56 [1.88-3.57], *q* < 0.0001), followed by 1,2-dipalmitoyl-GPC (16:0/16:0) (OR 2.46 [1.67-3.90], *q* = 0.0001), 1-stearoyl-2-arachidonoyl-GPC (18:0/20:4) (OR 2.37 [1.75-3.31], *q* < 0.0001), palmitoyl sphingomyelin (d18:1/16:0) (OR 2.19 [1.65-2.99], *q* < 0.0001) and sphingomyelin (d18:1/24:1, d18:2/24:0)∗ (OR 2.06 [1.57-2.77], *q* < 0.0001). Two metabolites, 4-ureidobutyrate (OR 0.71 [0.55-0.92], *q* = 0.017), and indolepropionylglycine (OR 0.77 [0.59-0.98], *q* = 0.045), were associated with lower odds of nondiabetic CKD. Moreover, there were no significant interactions between these urine metabolites and ethnicity.

The highest-ranked metabolites in the prediction of nondiabetic CKD showed only minor overlap between plasma and urine (Fig S4). In the assessment of the top 20 highest-ranked plasma metabolites, the carbohydrate glycerate also featured in the top 100 predictors for urine metabolites. Vice versa, in the top 20 highest-ranked urine metabolites, we found that hypoxanthine, phosphocholine, and glucoronate were also ranked among the top 100 predictors for plasma metabolites.

### Comparison Between CKD and DKD in Best Predicting Plasma and Urine Metabolites

Compared with participants with nondiabetic CKD, the 45 individuals with DKD were older, more frequently men and more often of African Surinamese and South Asian Surinamese origin, while having a higher BMI and prevalence of hypertension (Table S3). Although kidney filtration function was preserved in the DKD group, the eGFR of DKD subjects was significantly lower than controls (98.5 mL/min/1.73m^2^ for controls versus 84.3 mL/min/1.73m^2^ for DKD, *P* < 0.001). The UACR in the nondiabetic CKD group was 5.4 mg/mmol and in the DKD group 8.8 mg/mmol (*P* < 0.001).

**Figure 3:**
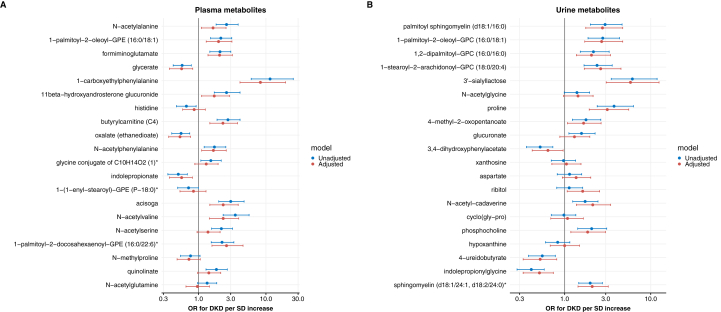
Logistic regression models: odds ratios (OR) per standard deviation (SD) increase for top 20 predictors of diabetic kidney disease (DKD) compared with healthy controls derived from (A) plasma metabolites and (B) urine metabolites. In the adjusted model (red), we corrected for age, sex, body mass index, and hypertension.

To assess whether the best predicting metabolites for nondiabetic CKD were similarly associated with DKD, we performed logistic regression models with these metabolites in a population of patients with DKD with the same group of control participants (Fig 3). Of the 20 highest-ranked plasma metabolites for nondiabetic CKD, 12 metabolites were also associated with DKD, such as N-acetylalanine, 1-palmitoyl-2-oleoyl-GPE (16:0/18:1), formiminoglutamate, glycerate, and 1-carboxyethylphenylalanine. Except for glycerate, the effect sizes of these plasma metabolites were larger for DKD than for nondiabetic CKD, especially for 1-carboxyethylphenylalanine (OR 8.28 [4.12-19.57] for DKD, *q* < 0.0001). Fourteen of the 20 highest-ranked urine metabolites for nondiabetic CKD were associated with DKD, including the 4 highest-ranked lipid metabolites. In general, the effect sizes of these urine lipids were larger in DKD compared with non-diabetic CKD. The carbohydrate 3’-sialyllactose (OR 5.82 [3.04-12.50], *q* < 0.0001) and the amino acid proline (OR 3.75 [2.93-6.30], *q* < 0.0001) had a much stronger association with DKD. In contrast, hypoxanthine was not associated with DKD while showing a clear association with non-diabetic CKD.

## Discussion

In this population-based cohort of more than 300 individuals with CKD and DKD with preserved kidney function and unimpaired metabolite clearance, we found that urine metabolites demonstrate stronger associations with nondiabetic CKD than plasma metabolites. Although the top-ranked plasma metabolites predominantly comprised amino acids, the top urine metabolites included many lipids. As a result, the plasma and urine metabolomics analyses showed limited overlap. There were no interactions with ethnicity, underscoring the robustness of our results across the different ethnic groups. Because the associations of these plasma and urine metabolites could be translated to subjects with DKD, this study shows for the first time that CKD and DKD have similar metabolite profiles, which is likely based on shared underlying pathophysiological mechanisms.

In our plasma metabolomics analyses, we found a range of amino acids that were associated with CKD, with N-acetylated amino acids being particularly more abundant in the CKD group compared with controls. In line, a study reported that *NAT8* variants (encoding hepatic and renal acetyltransferase) caused an increase in levels of N-acetylated amino acids, which were associated with an increased risk of kidney failure.^14^ Acetylation improves the water solubility of metabolites, facilitating their excretion. *NAT8* variants might alter acetylation processes and lead to accumulation of potentially toxic metabolites.^15^ N-acetylated amino acids, and in particular N-acetylserine, has been previously associated with CKD progression in the CRIC cohort.^16^

We observed higher phenylalanine levels to be associated with CKD, with an even stronger association with DKD.^17^^,^^18^ Phenylalanine, which can be placed in the catecholamine synthesis pathway, has been associated with hypertension, one of the main causes of nondiabetic CKD.^19^^,^^20^ Interestingly, the kidneys also play a significant role in phenylalanine metabolism, including the hydroxylation of phenylalanine to tyrosine, and the excretion of phenylalanine.^17^^,^^21^ As a result, CKD could cause an accumulation of plasma metabolites derived from both phenylalanine and tyrosine.

Histidine and the histidine-derivative formimoglutamine levels were both lower in individuals with nondiabetic CKD, in line with an observational study in patients with CKD that showed inverse associations of histidine levels with systemic inflammatory markers and subsequent mortality. Animal studies provided further evidence for the protective role of histidine supplementation against hypertension and CKD progression, which is attributed to its antioxidant properties.^22^^,^^23^ Other histidine metabolites were also associated with CKD progression in the CRIC study.^16^^,^^24^

In addition, we observed lower levels of indolepropionate, a microbial metabolite, in nondiabetic CKD. Indolepropionate, a product of tryptophan degradation, has recently been associated with lower cardiometabolic disease incidence in a longitudinal study, as opposed to other tryptophan metabolites, which were linked to increased incidence.^25^^,^^26^ In patients with CKD specifically, tryptophan levels have been associated with a higher incidence of cardiovascular disease.^27^^,^^28^ The mechanisms that explain the impact of indole-3-propionate on kidney damage remain to be uncovered.

Among the best predictors of the urine metabolites in machine learning model for nondiabetic CKD, we identified 2 sphingomyelins. This is in line with previous data showing that in CKD, sphingomyelins tend to accumulate within the glomerular cells, subsequently causing glomerular proliferation and hypertrophy.^29^ Ceramide has also been strongly related to whole-body insulin resistance.^30^ As kidneys are metabolically very active tissue due to high adenosine triphosphate demand to facilitate sodium reabsorption, insulin resistance has been linked to inefficient substrate metabolism, rendering the kidneys at risk for hypoxia.^31^

We also found several phosphatidylcholines and phosphocholine to be strongly associated with both CKD and DKD. Phosphatidylcholine metabolites in urine have also been associated with adverse kidney outcomes in (mixed etiology) CKD in the German CKD study.^32^ There are diverse pathways through which these metabolites could be involved in conferring kidney damage. Higher levels of these metabolites may be a reflection of dietary phosphocholine intake. Phosphocholine can be converted to trimethylamine by the gut microbiota, which is subsequently metabolized by the liver into trimethylamine oxide (TMAO).^33^^,^^34^ Elevated TMAO levels have been associated with vascular inflammation and increased thrombogenicity.^35^, ^36^, ^37^ Alternatively, lysophosphatidylcholine (LPC), derived from phosphatidylcholine, could play a role in the association with CKD. Cellular stress could cause accumulation of lysophosphatidylcholine in the kidney, inducing apoptosis due to its lipotoxic nature and further aggravating kidney damage.^38^^,^^39^

Overall, most of the retrieved associations with nondiabetic CKD could be translated to DKD, yet not all metabolites were similarly associated in the strength of their association with both conditions. For instance, the association between xanthosine levels and nondiabetic CKD did not translate to DKD. The association with xanthosine and cardiorenal disease is in line with the existing literature, including findings of the Framingham Heart Study and animal models of CKD.^40^^,^^41^ Xanthosine, a purine nucleoside, can be metabolized in xanthine and uric acid, respectively. A higher excretion of xanthosine pathway metabolites has been associated with conditions of increased oxidative stress, such as acute coronary syndromes.^42^ Because uric acid excretion tends to be higher in patients on angiotensin converting enzyme inhibitors, and the use of this medication was higher in the DKD subjects in this cohort, this could explain the lack of association between xanthosine and DKD. Conversely, the association of 3’-siallylactose with DKD was much stronger than with nondiabetic CKD. Sialyllactose is a human milk oligosaccharide that consists of sialic acid bound to lactose. Indeed, sialic acid levels were reported to be specifically higher in people living with diabetes and were also reported to be indicative of microvascular damage associated with this disease.^43^

Our study has several limitations. First, our analyses were conducted using a cross-sectional data, and as a result, we cannot evaluate whether the metabolites identified as the most predictive are indicative of pathological or protective mechanisms. We did not have data on underlying causes of CKD in this population-based cohort, which could have provided more mechanistic insight. In addition, the classification model to predict nondiabetic CKD from plasma metabolite profiles yielded only an AUC of 0.59, and therefore, results should be interpreted with caution. In the urine metabolomics analyses, we adjusted for creatinine levels to account for differences in urine concentration. However, the urine metabolites were measured in a morning spot sample, which might not be reflective of 24 hours excretion. Finally, differences in diet and circadian rhythms may have introduced additional variation. Strengths of this study include the large sample size and availability of both plasma and urine metabolomics in a multiethnic cohort. Although we did not find differences between ethnicities when testing interactions, the inclusion of multiple ethnic groups contributes to the external validity of our findings. Our untargeted analysis sheds light on potential mechanisms underlying nondiabetic CKD that could also be extended to a DKD population.

In conclusion, we showed that urine metabolites are more strongly associated with nondiabetic CKD than plasma metabolites. Furthermore, we demonstrate the predictive potential of a range of metabolites for both nondiabetic CKD and DKD, supporting the notion of shared pathogenic drivers between the 2 conditions. With the advent of novel kidney-protective drugs such as sodium glucose cotransporter-2 inhibitors, mineralocorticoid receptor antagonists, and glucagon-like peptide-1 receptor agonists, all with different kidney-protective mechanisms of action, treatment based on biomarkers to choose an optimal therapy, has been proposed be the future of clinical kidney care. Analyses such as ours may provide a first step in stratification of patients, especially when different cohorts are combined in future studies. From a mechanistic perspective, the identification of metabolites that can be further investigated in vitro, may lead to recognition of novel pathogenic pathways that pave the way for novel treatment avenues.

## Supplementary Materials

Supplementary File (PDF) Figure S1-S4; Item S1; Tables S1-S3.
