# Supplementary material for: Plasma and Urine Metabolites Associated With Nondiabetic Chronic Kidney Disease: The HELIUS Study
Source: Kidney Med. 2025 Apr 17;7(7):101009. doi: 10.1016/j.xkme.2025.101009 (PMC12221752; doi:10.1016/j.xkme.2025.101009)
Supplement: Supplementary File (PDF) — Figure S1-S4; Item S1; Tables S1-S3. [file mmc1.pdf]

Item S1: Supplementary Methods

Source: Supplementary Methods of UPLC-MS/MS as provided by Metabolon.

Sample shipment and processing

All samples were kept at -80°C until processed. Samples were prepared using the automated MicroLab STAR® system (Hamilton Company). Several recovery standards were added prior to the first step in the extraction process for QC purposes. To remove protein, dissociate small molecules bound to protein or trapped in the precipitated protein matrix, and to recover chemically diverse metabolites, proteins were precipitated with methanol under vigorous shaking for 2 min (Glen Mills GenoGrinder 2000) followed by centrifugation. The resulting extract was divided into five fractions: two for analysis by two separate reverse phase (RP)/UPLC-MS/MS methods with positive ion mode electrospray ionization (ESI), one for analysis by RP/UPLC-MS/MS with negative ion mode ESI, one for analysis by HILIC/UPLC-MS/MS with negative ion mode ESI, and one sample was reserved for backup. Samples were placed briefly on a Zymark TurboVap® evaporator to remove the solvent. The sample extracts were stored in nitrogen overnight before preparation for analysis.

QA/QC

Several types of controls were analyzed together with the study plasma samples: a pooled matrix sample generated by taking a small volume of each experimental sample served as a technical replicate throughout the data set; extracted water samples served as process blanks; and a cocktail of QC standards that were carefully chosen not to interfere with the measurement of endogenous compounds were spiked into every analyzed sample, allowed instrument performance monitoring and aided chromatographic alignment. Tables 1 and 2 describe these QC samples and standards. Instrument variability was determined by calculating the median relative standard deviation (RSD) for the standards that were added to each sample prior to injection into the mass spectrometers. Overall process variability was determined by calculating the median RSD for all endogenous metabolites (i.e., non-instrument standards) present in 100% of the pooled matrix samples. Experimental samples were randomized across the platform run with QC samples spaced evenly among the injections, as outlined in Figure 1.

Table 1: Description of Metabolon QC Samples

Type	Description	Purpose
MTRX	Large pool of human plasma maintained by Metabolon that has been characterized extensively.	Assure that all aspects of the Metabolon process are operating within specifications.
CMTRX	Pool created by taking a small aliquot from every customer sample.	Assess the effect of a non-plasma matrix on the Metabolon process and distinguish biological variability from process variability.
PRCS	Aliquot of ultra-pure water	Process Blank used to assess the contribution to compound signals from the process.
SOLV	Aliquot of solvents used in extraction.	Solvent Blank used to segregate contamination sources in the extraction.

Table 2: Metabolon QC Standards

Type	Description	Purpose
RS	Recovery Standard	Assess variability and verify performance of extraction and instrumentation.
IS	Internal Standard	Assess variability and performance of instrument.

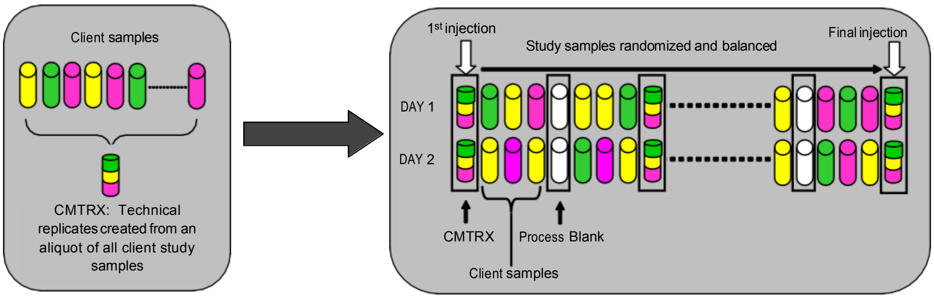

Figure 1. Preparation of client-specific technical replicates. A small aliquot of each client sample (colored cylinders) is pooled to create a CMTRX technical replicate sample (multi-colored cylinder), which is then injected periodically throughout the platform run. Variability among consistently detected biochemicals can be used to calculate an estimate of overall process and platform variability.

Ultrahigh Performance Liquid Chromatography-Tandem Mass Spectroscopy (UPLC-MS/MS)
All UPLC-MS/MS methods used a Waters ACQUITY UPLC and a Thermo Scientific Q-Exactive high resolution/accurate mass spectrometer interfaced with a heated electrospray ionization (HESI-II) source and Orbitrap mass analyzer operated at 35,000 mass resolution. The sample extract was dried then reconstituted in solvents compatible to each of the four methods. Each reconstitution solvent contained a series of standards at fixed concentrations to ensure injection and chromatographic consistency. One aliquot was analyzed using acidic positive ion conditions, chromatographically optimized for more hydrophilic compounds. In this method, the extract was gradient eluted from a C18 column (Waters UPLC BEH C18-2.1x100 mm, 1.7 µm) using water and methanol, containing 0.05% perfluoropentanoic acid (PFPA) and 0.1% formic acid (FA). Another aliquot was also analyzed using acidic positive ion conditions, however it was chromatographically optimized for more hydrophobic compounds. In this method, the extract was gradient eluted from the same afore mentioned C18 column using methanol, acetonitrile, water, 0.05% PFPA and 0.01% FA and was operated at an overall higher organic content. Another aliquot was analyzed using basic negative ion optimized conditions using a separate dedicated C18 column. The basic extracts were gradient eluted from the column using methanol and water, however with 6.5mM Ammonium Bicarbonate at pH 8. The fourth aliquot was analyzed via negative ionization following elution from a HILIC column (Waters UPLC BEH Amide 2.1x150 mm, 1.7 µm) using a gradient consisting of water and acetonitrile with 10mM Ammonium Formate, pH 10.8. The MS analysis alternated between MS and data-dependent MS^n scans using dynamic exclusion. The scan range varied slighted between methods but covered 70-1000 m/z. Raw data files are archived and extracted as described below.

Bioinformatics
The informatics system consisted of four major components, the Laboratory Information Management System (LIMS), the data extraction and peak-identification software, data processing tools for QC and compound identification, and a collection of information interpretation and visualization tools for use by data analysts. The hardware and software foundations for these informatics components were the LAN backbone, and a database server running Oracle 10.2.0.1 Enterprise Edition.

Data Extraction and Compound Identification
Raw data was extracted, peak-identified and QC processed using Metabolon's hardware and software. These systems are built on a web-service platform utilizing Microsoft's .NET technologies, which run on high-performance application servers and fiber-channel storage arrays in clusters to provide active failover and load-balancing. Compounds were identified by comparison to library entries of purified standards or recurrent unknown entities. Metabolon maintains a library based on authenticated standards that contains the retention time/index (RI), mass to charge ratio (*m/z)*, and chromatographic data (including MS/MS spectral data) on all molecules present in the library. Furthermore, biochemical identifications are based on three criteria: retention index within a narrow RI window of the proposed identification, accurate mass match to the library ±10 ppm, and the MS/MS forward and reverse scores between the experimental data and authentic standards. The MS/MS scores are based on a comparison of the ions present in the experimental spectrum to the ions present in the library

spectrum. While there may be similarities between these molecules based on one of these factors, the use of all three data points can be utilized to distinguish and differentiate biochemicals. More than 3300 commercially available purified standard compounds have been acquired and registered into LIMS for analysis on all platforms for determination of their analytical characteristics. Additional mass spectral entries have been created for structurally unnamed biochemicals, which have been identified by virtue of their recurrent nature (both chromatographic and mass spectral). These compounds have the potential to be identified by future acquisition of a matching purified standard or by classical structural analysis.

Curation

A variety of curation procedures were carried out to ensure that a high-quality data set was made available for statistical analysis and data interpretation. The QC and curation processes were designed to ensure accurate and consistent identification of true chemical entities, and to remove those representing system artifacts, mis-assignments, and background noise. Metabolon data analysts use proprietary visualization and interpretation software to confirm the consistency of peak identification among the various samples. Library matches for each compound were checked for each sample and corrected if necessary. Peaks were quantified using area-under-the-curve.

Metabolite Quantification and Data Normalization

Peaks were quantified using area-under-the-curve. For studies spanning multiple days, a data normalization step was performed to correct variation resulting from instrument inter-day tuning differences. Essentially, each compound was corrected in run-day blocks by registering the medians to equal one (1.00) and normalizing each data point proportionately (termed the "block correction").

Supplementary Tables

Table S1: Plasma metabolites logistic regression models

Metabolite	m0-est	m0-l95	m0-u95	m0-q	m1-est	m1-l95	m1-u95	m1-q
N-acetylalanine	1.71	1.34	2.23	<0.001	1.51	1.16	2.00	0.006
1-palmitoyl-2-oleoyl-GPE (16:0/18:1)	1.71	1.35	2.21	<0.001	1.62	1.27	2.11	0.003
formiminoglutamate	1.49	1.19	1.89	0.001	1.44	1.13	1.85	0.007
glycerate	0.6	0.47	0.75	<0.001	0.63	0.49	0.8	0.003
1-carboxyethylphenylalanine	1.62	1.27	2.07	<0.001	1.45	1.12	1.9	0.010
11beta-hydroxyandrosterone glucuronide	1.28	1.01	1.63	0.047	1.31	1.00	1.75	0.064
histidine	0.66	0.52	0.84	0.001	0.67	0.52	0.86	0.005
butyrylcarnitine (C4)	1.61	1.27	2.08	<0.001	1.5	1.17	1.96	0.005
oxalate (ethanedioate)	0.61	0.48	0.77	<0.001	0.63	0.49	0.81	0.003
N-acetylphenylalanine	1.53	1.21	1.96	0.001	1.46	1.14	1.9	0.007
glycine conjugate of C10H14O2 (1)*	1.36	1.08	1.72	0.013	1.33	1.04	1.7	0.029
indolepropionate	0.68	0.53	0.86	0.003	0.75	0.58	0.95	0.028
1-(1-enyl-stearoyl)-GPE (P-18:0)*	0.95	0.75	1.19	0.636	0.98	0.77	1.24	0.860
acisoga	1.44	1.14	1.84	0.004	1.43	1.11	1.85	0.010
N-acetylvaline	1.72	1.34	2.25	<0.001	1.59	1.21	2.13	0.005
N-acetylserine	1.65	1.29	2.16	<0.001	1.52	1.17	2.01	0.006
1-palmitoyl-2-docosahexaenoyl-GPE (16:0/22:6)*	1.6	1.27	2.06	<0.001	1.52	1.18	1.98	0.005
N-methylproline	0.76	0.6	0.95	0.021	0.74	0.58	0.94	0.023
quinolinate	1.19	0.95	1.5	0.147	0.97	0.76	1.25	0.860
N-acetylglutamine	1.44	1.14	1.84	0.004	1.33	1.04	1.73	0.031

Model 0 is unadjusted; model 1 is adjusted for age, sex, BMI and hypertension. Est = estimate (odds ratio), l95 = lower 95% confidence interval, u95 = upper 95% confidence interval. q-value = adjusted p-value using false discovery rate.

Table S2: Urine metabolites logistic regression models

Metabolite	m0-est	m0-l95	m0-u95	m0-q	m1-est	m1-l95	m1-u95	m1-q
palmitoyl sphingomyelin (d18:1/16:0)	1.98	1.52	2.63	<0.001	2.19	1.65	2.99	<0.001
1-palmitoyl-2-oleoyl-GPC (16:0/18:1)	2.27	1.72	3.09	<0.001	2.56	1.88	3.57	<0.001
1,2-dipalmitoyl-GPC (16:0/16:0)	2.25	1.58	3.45	<0.001	2.46	1.67	3.9	<0.001
1-stearoyl-2-arachidonoyl-GPC (18:0/20:4)	2.21	1.67	3.01	<0.001	2.37	1.75	3.31	<0.001
3'-sialyllactose	1.36	1.08	1.75	0.018	1.3	1.01	1.69	0.051
N-acetylglycine	1.13	0.9	1.41	0.302	1.13	0.89	1.44	0.311
proline	1.55	1.22	2	0.001	1.65	1.27	2.18	0.001
4-methyl-2-oxopentanoate	1.56	1.23	2.01	0.001	1.67	1.28	2.22	0.001
glucuronate	1.3	1.03	1.65	0.040	1.41	1.09	1.84	0.017
3,4-dihydroxyphenylacetate	0.79	0.63	0.99	0.055	0.87	0.68	1.11	0.278
xanthosine	1.32	1.05	1.67	0.028	1.42	1.1	1.85	0.014
aspartate	1.58	1.25	2.02	0.001	1.66	1.29	2.17	<0.001
ribitol	1.18	0.94	1.5	0.169	1.28	0.99	1.68	0.072
N-acetyl-cadaverine	1.54	1.22	1.97	0.001	1.6	1.24	2.08	0.001
cyclo(gly-pro)	1.22	0.97	1.54	0.098	1.33	1.03	1.73	0.040
phosphocholine	1.4	1.11	1.79	0.009	1.38	1.07	1.79	0.020
hypoxanthine	1.3	1.03	1.65	0.040	1.46	1.13	1.92	0.010
4-ureidobutyrate	0.74	0.59	0.93	0.018	0.71	0.55	0.92	0.017
indolepropionylglycine	0.69	0.54	0.88	0.006	0.77	0.59	0.98	0.046
sphingomyelin (d18:1/24:1, d18:2/24:0)*	1.77	1.4	2.28	<0.001	2.06	1.57	2.77	<0.001

Model 0 is unadjusted; model 1 is adjusted for age, sex, BMI and hypertension. Est = estimate (odds ratio), l95 = lower 95% confidence interval, u95 = upper 95% confidence interval. q-value = adjusted p-value using false discovery rate.

Table S3: Population characteristics: controls and DKD

	Controls	DKD	*p-value*
**n**	200	45	
**Age in years**	49.6±11.4	60.2±6.0	<0.001
**Women**	109 (54.5)	19 (42.2)	0.185
**Ethnicity**			<0.001
Dutch	50 (25.0)	0 (0.0)	
South-Asian Surinamese	50 (25.0)	27 (60.0)	
African Surinamese	50 (25.0)	18 (40.0)	
Ghanaian	50 (25.0)	0 (0.0)	
**BMI in kg/m^2**	26.2±4.5	30.1±5.2	<0.001
**Current smoking**	33 (16.6)	10 (22.2)	0.496
**Cardiovascular disease**	20 (10.0)	9 (20.0)	0.105
**Hypertension**	89 (44.5)	39 (86.7)	<0.001
**Blood pressure lowering drugs**	40 (20.0)	28 (62.2)	<0.001
**Systolic BP in mmHg**	130.0±17.7	148.4±22.2	<0.001
**Diastolic BP in mmHg**	81.03 (10.24)	83.90 (9.42)	0.086
**eGFR in ml/min**	98.5±17.1	84.3±23.4	<0.001
**UACR in mg/mmol**	0.25 [0.15, 0.41]	8.78 [5.88, 13.68]	<0.001
**HbA1C mmol/mol**	37.6±4.6	58.2±15.7	<0.001
**Total cholesterol in mmol/L**	5.1±0.9	4.4±1.1	<0.001
**LDL in mmol/L**	3.2±0.8	2.6±0.9	<0.001
**Triglycerides in mmol/L**	0.72 [0.57, 1.01]	1.25 [0.88, 1.76]	<0.001
**Framingham risk score**	7.9 [3.3, 13.2]	30.0 [18.5, 30.0]	<0.001

Data is presented as mean±SD, median[IQR] or n (%). Group differences were tested with t-test for continuous variables with normal distribution, Mann-Whitney U for continuous variables with non-normal distribution, and chi-square tests for categorical variables. BMI = body mass index, BP = blood pressure, eGFR = estimated glomerular filtration rate (CKD-EPI), UACR = urine albumin creatinine ratio, LDL = low-density lipoprotein.

Supplementary Figures

Figure S1: Participant flowchart

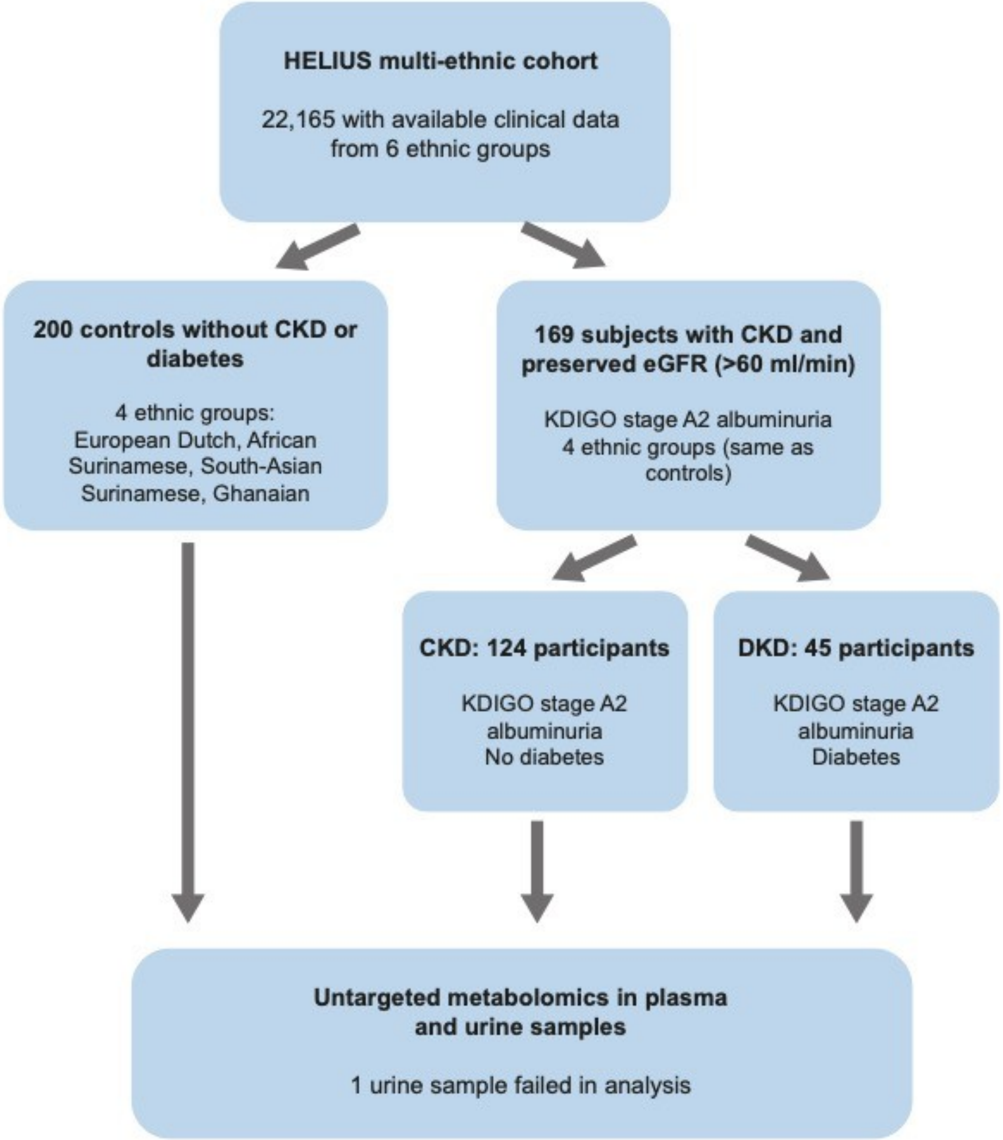

Figure S2: Plasma metabolite feature importance

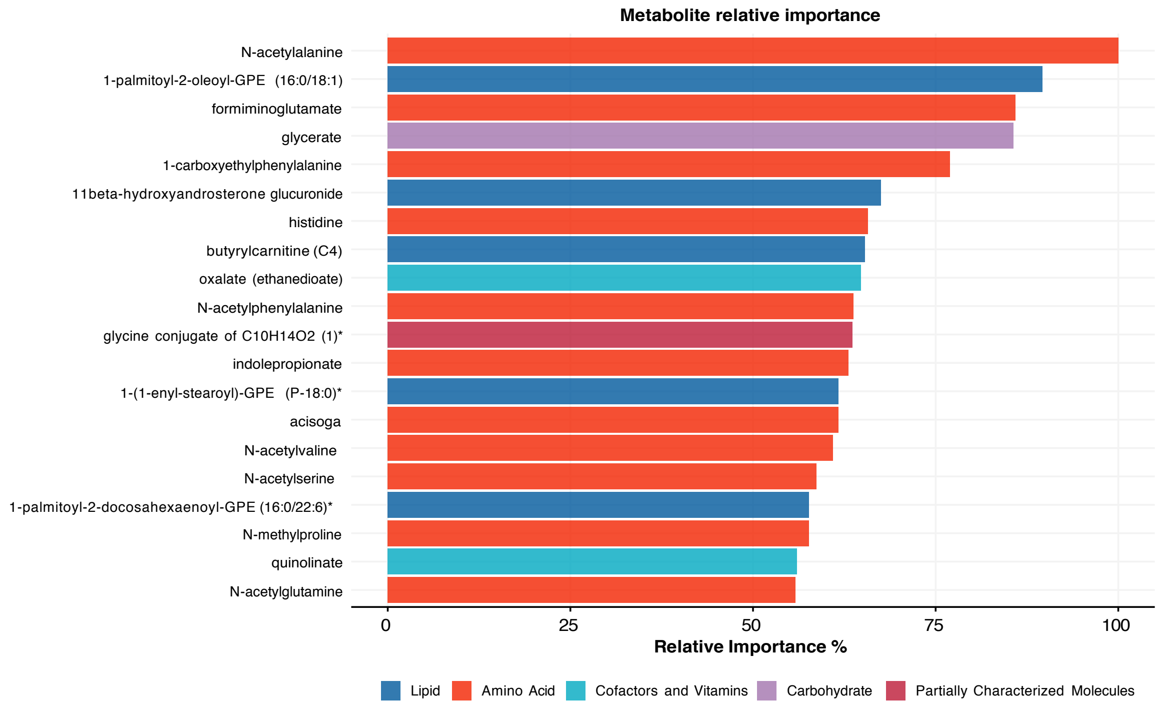

Relative feature importance of top 20 highest ranked plasma metabolites in the prediction for non-diabetic CKD. The feature importance was calculated using the average gain of features to the XGBoost model.

Figure S3: Urine metabolites feature importance

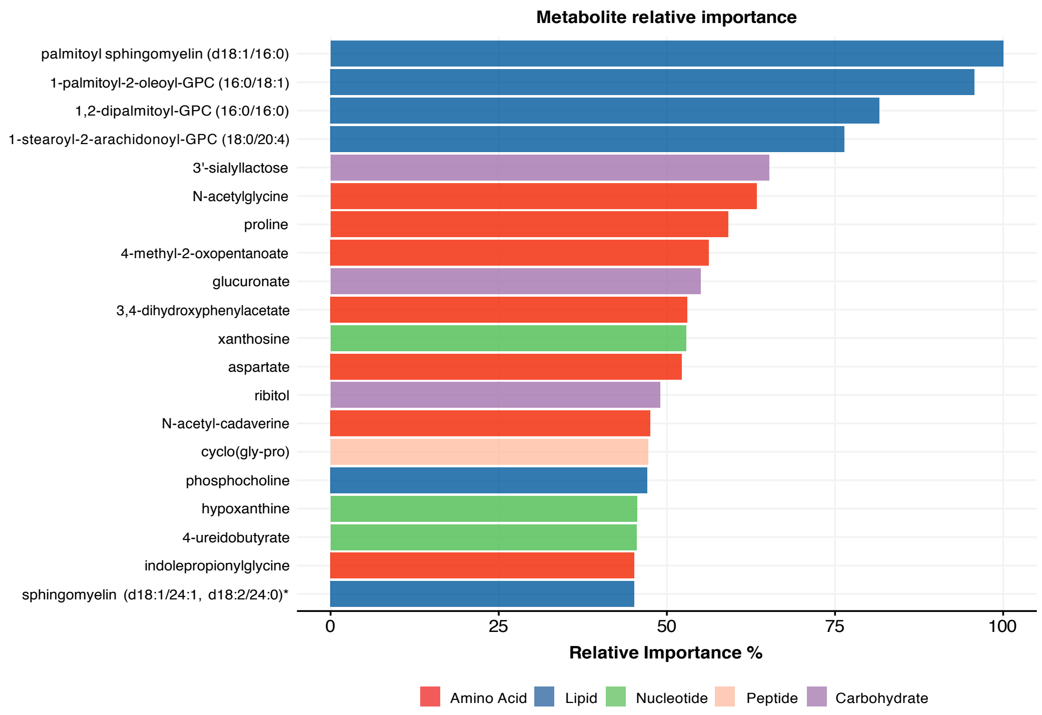

Relative feature importance of top 20 highest ranked urine metabolites in the prediction for non-diabetic chronic kidney disease (CKD). The feature importance was calculated using the average gain of features to the XGBoost model.

Figure S4: Overlap plasma and urine metabolites

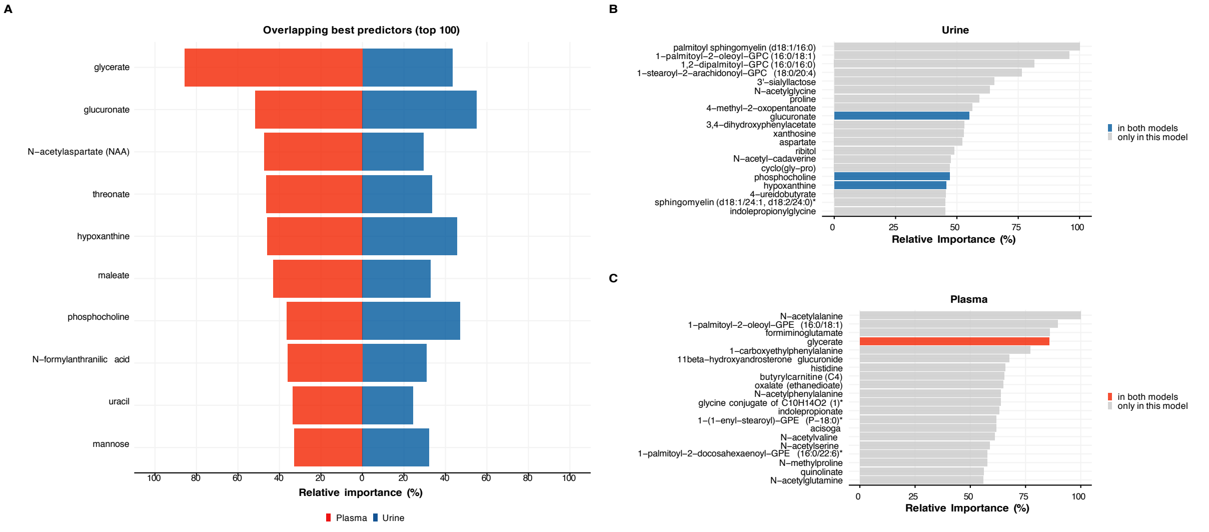

Figure B and C show the top 20 predictors; these are marked as "in both models" if this predictor appears in the top 100 predictors of the other category (plasma or urine)

Overlap between plasma and urine best predicting metabolites for non-diabetic CKD. A. The 10 overlapping metabolites in the top 100 for non-diabetic CKD. B. The top 20 urine metabolites; highlighted metabolites also appeared in the top 100 of plasma metabolites. C. The top 20 plasma metabolites; highlighted metabolites also appeared in the top 100 of urine metabolites.
